# A More Accessible, Time-Saving, and Efficient Method for In Vitro Plant Regeneration from Potato Protoplasts

**DOI:** 10.3390/plants10040781

**Published:** 2021-04-16

**Authors:** Ki-Beom Moon, Ji-Sun Park, Su-Jin Park, Hyo-Jun Lee, Hye-Sun Cho, Sung-Ran Min, Youn-Il Park, Jae-Heung Jeon, Hyun-Soon Kim

**Affiliations:** 1 Plant Systems Engineering Research Center, Korea Research Institute of Bioscience and Biotechnology, 125 Gwahak-ro, Yuseong-gu, Daejeon 34141, Korea; irony83@kribb.re.kr (K.-B.M.); pjs12315@kribb.re.kr (J.-S.P.); tnwls@kribb.re.kr (S.-J.P.); hyojunlee@kribb.re.kr (H.-J.L.); hscho@kribb.re.kr (H.-S.C.); srmin@kribb.re.kr (S.-R.M.); 2 Department of Biosystems and Bioengineering, University of Science & Technology, 217 Gajung-ro, Yuseong-gu, Daejeon 34113, Korea; 3 Department of Biological Sciences, Chungnam National University, 99 Deahank-ro, Yuseong-gu, Daejeon 34134, Korea; yipark@cnu.ac.kr

**Keywords:** *Solanum tuberosum*, isolation, purification, alginate lens, callus greening

## Abstract

Both obtaining high-yielding, viable protoplasts and following reliable regeneration protocols are prerequisites for the continuous expansion and development of newly emerging systems involving protoplast utilization. This study determines an efficient process from protoplast isolation to shoot regeneration in vitro. The maximum yield of protoplast extraction, which was 6.36 ± 0.51 × 10^6^ protoplasts/g fresh weight (FW), was approximately 3.7 times higher than that previously reported for potato protoplasts. To obtain data, wounded leaves were used by partially cutting both sides of the midrib, and isolated protoplasts were purified by the sucrose cushion method, with a sucrose concentration of 20%. We confirmed a significant effect on the extraction efficiency by measuring enzymolysis during a 6 h period, with three times more washing buffer than the amount normally used. Protoplasts fixed in alginate lenses with appropriate space were successfully recovered and developed into microcalli 2 weeks after culture. In addition, to induce high efficiency regeneration from protoplasts, calli in which greening occurred for 6 weeks were induced to develop shoots in regeneration medium solidified by Gelrite, and they presented a high regeneration efficiency of 86.24 ± 11.76%.

## 1. Introduction

Since 1975 [1], potato protoplast cultures have been studied by researchers worldwide to establish efficient and reproducible methods for shoot regeneration. The purpose of most of these studies is to generate phenotypic variation by the use of protoplast-derived variable mutants or clonal propagation with karyotype stability [2,3]. In particular, in vegetatively propagated plant species such as potato, plant improvement via protoplast culture methods is considered [4,5]. Since the 1980s, protoplasts have been treated as a model material for somaclonal variation, and the use of protoplasts as an alternative to traditional cross-breeding for commercial purposes has received increasing amounts of attention. To date, varietal improvements, such as non-browning, heat tolerance, increased resistance, improved starch content and yield, and increased nutrient and antioxidant contents in potato, have been achieved through somaclonal variation [6,7,8,9,10]. Another significant field in which protoplasts are used is the development of novel genotypes via somatic fusions. Somatic hybrid potato and tomato plants have been developed from fused protoplasts [11]. More recently, protoplasts of *Lycopersicon pennellii* Corr., a wild relative of tomato, were electrofused with those of a dihaploid potato clone (cultivar Nicola), with the objective of transferring salinity tolerance from *L. pennellii* to cultivated potato [12], and bacterial wilt-resistant somatic hybrids have been obtained via protoplast fusion between potato and eggplant [13]. Specifically, interspecific somatic hybridization in potato was developed by protoplast electrofusion to transfer insect resistance [14,15], disease resistance [16], metribuzin tolerance [17], and late blight resistance [18].

Potato is one of several crop species that can easily be transformed with foreign genes by *Agrobacterium* and that produces a high number of transgenic shoots in a short time period, and new varieties have been developed mainly by this method since 1980 [19]. The recently developed DNA-free genome editing technology in plants enables editing of desired sites without transgene genome integration by introducing ribonucleoproteins (RNPs) into plant protoplasts [20,21]. RNPs-mediated genome editing technology appears to be a very useful tool for plant breeding. Woo et al. reported that RNPs were successfully inserted into the protoplasts of lettuce, resulting in edited lettuce plants [22]. However, the efficiency of RNPs delivery into protoplasts by polyethylene glycol (PEG)-mediated transfection or by biolistics and plant regeneration from edited protoplasts is still low. To improve the success of the development of genome edited plants, a high and stable level of protoplast-derived plant regeneration efficiency must be achieved. Many studies have reported optimal conditions for all stages/steps, from protoplast extraction to plant regeneration. For example, a high protoplast extraction efficiency of 4.60 × 10^6^/g of calli and a maximum germination frequency of 81% were shown in coriander varieties [23]. On the other hand, the protoplast extraction efficiency of 2 × 10^5^ per g of leaf tissue was shown in *Dalbergia sissoo*. [24]. According to these studies, the highest efficiency so far, especially with respect to potato genome editing, is 9% (RNPs using synthesized gRNA), and reproducibility varies on a case-by-case basis [25].

In this study, we aimed to achieve a high regeneration rate of in vitro shoots from protoplasts by checking and controlling several factors based on previously reported methods, such as isolation, purification, callus induction, and regeneration. The results reveal that material preparation, enzymolysis conditions, alginate fixation, and medium and culture periods for callus greening affect regeneration efficiency, and fine tuning these factors can lead to time savings and easy plant regeneration.

## 2. Results

### 2.1. Isolation Efficiency of Protoplasts Depends on the Preparation Conditions of the Plant Material

**Figure 1:**
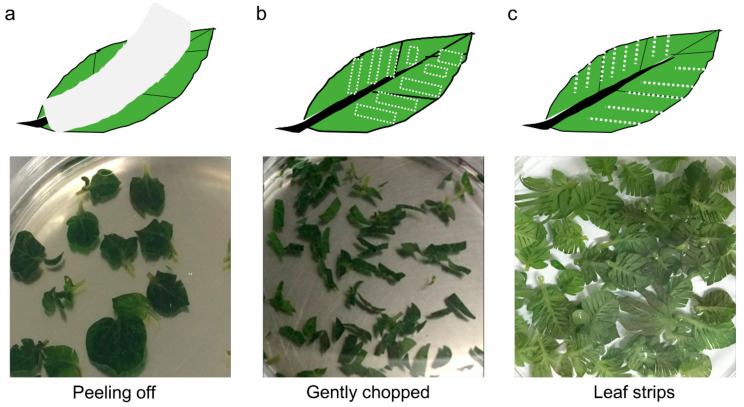
Three different methods of leaf wounding. (**a**) Both sides of the epidermis were adhered to clear 3M tape, and the lower epidermal layer was removed by carefully pulling away the 3M tape. (**b**) Leaves were gently chopped into approximately 0.2 × 0.7 cm^2^ segments. (**c**) Partial wounds were made by removing 2–3 mm leaf strips on both sides of the midrib with a sterile blade.

To determine the most appropriate wounding method for protoplast extraction, the extraction efficiency of the three wounding methods was compared (Table 1). Epicuticular wax, which is a coating that covers the outer surface of the plant cuticle, was removed to induce surface wounds by attaching tape onto the leaf surface (Figure 1a). The efficiency of protoplast extraction was 4.45 ± 1.23 × 10^5^ protoplasts/g fresh weight (FW), and this result was the lowest compared to the results of the other two methods (Table 1). In addition, this method has its disadvantages: the experimental process involving tape attachment is cumbersome, and the potential for contamination is high. Wounding by gentle chopping (Figure 1b) was experimentally easier to perform, and the protoplast extraction efficiency was higher (7.14 ± 2.38 × 10^5^ protoplasts/g FW) than that of the leaf peeling method. Here, the method of partially cutting both sides of the leaf midrib (Figure 1c) yielded the highest protoplast extraction efficiency among the three methods tested (13.23 ± 2.8 × 10^5^ protoplasts/g FW), despite the small area of contact with the enzyme extraction solution and leaf cleavage site.

**Figure 2:**
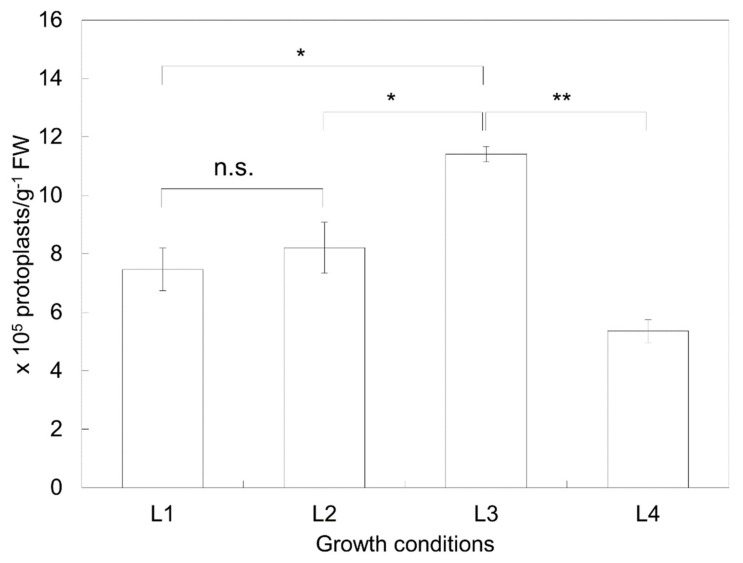
Effects of the physiological state of donor plant material on the extraction efficiency of protoplasts. Leaves were harvested from the upper first to third nodes of 2-week-old in vitro-grown plants (L1), 3-week-old in vitro-grown plants (L2), and 4-week-old in vitro-grown plants (L4). Leaves were harvested from plants grown in vitro for 2 weeks from axillary buds induced for 1 week (L3). *p*-values were obtained using Student’s *t*-test. *, *p* < 0.01; **, *p* < 0.0001; n.s., *p* > 0.05.

The effects of the physiological status of the plant material on the extraction efficiency were analyzed. The extraction efficiencies of protoplasts from the leaves of the 2-week-old in vitro-grown plants (L1), 3-week-old in vitro-grown plants (L2), 4-week-old in vitro-grown plants (L4), and 2-week-old in vitro-grown plants after 1 week of node culture (L3) were 7.467 ± 0.729 × 10^5^ protoplasts/g FW, 8.207 ± 0.870 × 10^5^ protoplasts/g FW, 11.407 ± 0.260 × 10^5^ protoplasts/g FW, and 5.360 ± 0.394 × 10^5^ protoplasts/g FW, respectively (Figure 2). The L3 material exhibited the best results, with an extraction efficiency that was approximately twice as high as the lowest value (which occurred for L4).

### 2.2. The Isolation Efficiency of Protoplasts Depends on the Extraction Conditions

The duration of incubation in the enzyme solution containing cellulase and macerozymes, called the enzymolysis time, affected the extraction efficiency of protoplasts. To optimize the enzymolysis time, leaves were digested for 5, 18, and 24 h, and the resulting isolation efficiencies were 5.24 ± 1.90 × 10^5^ protoplasts/g FW, 14.20 ± 2.96 × 10^5^ protoplasts/g FW, and 9.38 ± 0.88 × 10^5^ protoplasts/g FW, respectively. When the explants were incubated in the enzyme solution for 5 h, most of the extracted protoplasts were viable, but the amount of the extracted protoplasts was too low. On the other hand, when the time was increased to 24 h, the viability of the protoplasts decreased by 60% compared to that at 18 h, the latter of which was determined to be the best enzymolysis time (Table 2).

Four different factors—the ratio of cellulase and macerozymes, the volume of wash solution, the use of shaking, and the occurrence of plasmolysis—were randomly combined based on our preliminary experimental results, which yielded three experimental groups. From the above experiment (the data of which are shown in Table 2), we chose 18 h for the optimal enzymolysis time. Nevertheless, because time reduction is an important factor for efficient protoplast extraction, we tried to determine the optimal time through proper combinations with other experimental conditions (Table 3). When enzymolysis was applied for 5 h, 6 h, and 18 h under M1 conditions, the extraction efficiencies of the protoplasts were 5.24 ± 1.90 × 10^5^ protoplasts/g FW, 8.56 ± 1.84 × 10^5^ protoplasts/g FW, and 12.27 ± 3.40 × 10^5^ protoplasts/g FW, respectively. When enzymolysis was applied for 5 h, 6 h, and 18 h under M2 conditions, the efficiencies were 21.09 ± 8.49 × 10^5^ protoplasts/g FW, 39.63 ± 4.09 × 10^5^ protoplasts/g FW, and 28.14 ± 3.34 × 10^5^ protoplasts/g FW, respectively. When the extraction durations were 5 h and 6 h under M3 conditions, the efficiencies were 36.40 ± 4.66 × 10^5^ protoplasts/g FW and 63.59 ± 5.14 × 10^5^ protoplasts/g FW, respectively. When the results for each treatment time under different extraction conditions were compared, there was no significant difference between 6 h and 18 h under M1 conditions, but the values were 2.3 times lower at 5 h than at 18 h. Under M2 conditions, the highest efficiency reached 39.63 ± 4.09 × 10^5^ protoplasts/g FW, which was observed under the 6 h treatment, and this value was 1.38 times higher than that under the 18 h treatment. We therefore focused on the results from the M3 conditions. The M3 conditions were different from the M2 conditions during the protoplast washing process. Specifically, the extracted protoplasts were washed with a three-fold increased solution volume compared to that of the M1 and M2 conditions. As a result, we confirmed an obvious effect. After 6 h of treatment, the extraction efficiency was 39.63 ± 4.09 × 10^5^ protoplasts/g FW under M2 conditions, whereas it was 63.59 ± 5.14 × 10^5^ protoplasts/g FW under M3 conditions; this is an increase of approximately 146%. The reason for the high efficiency of protoplast extraction under M3 conditions is that the extraction buffer residue, which included the enzymes, was completely removed due to the addition of a large amount of washing solution; as a result, the enzymatic reaction was also completely stopped, which helped facilitate complete cell recovery.

### 2.3. The Purification of High-Quality Protoplasts Was Accomplished by the Use of a Sucrose Gradient

**Figure 3:**
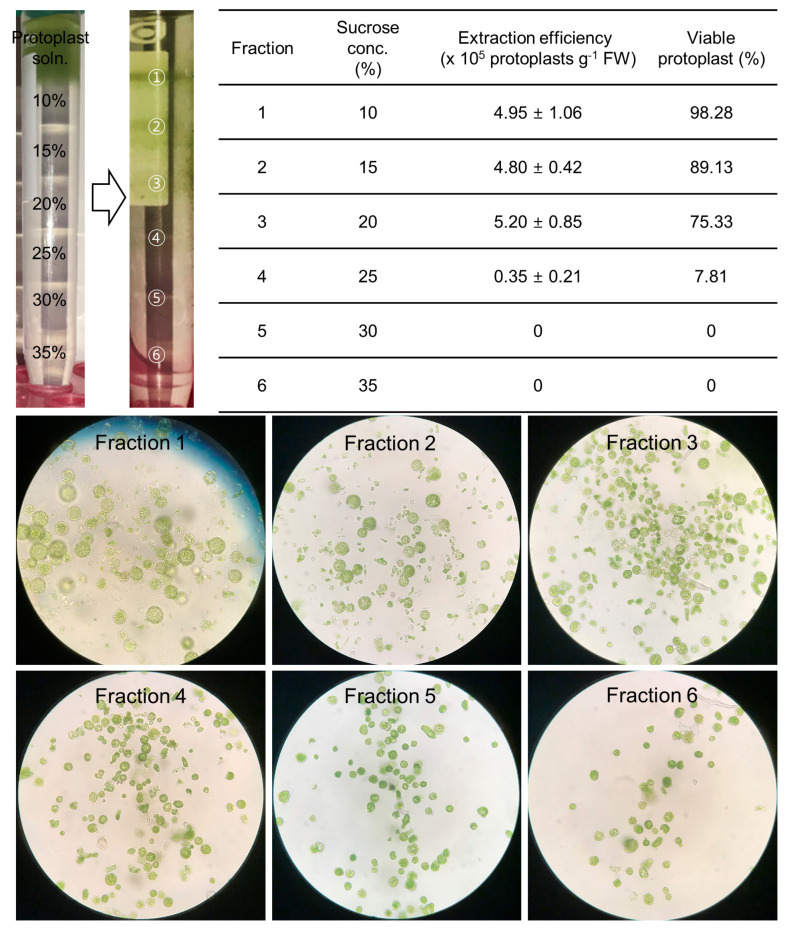
Comparison of protoplast extraction efficiencies by a sucrose gradient experiment involving 10–35% sucrose concentrations. Two milliliters of each solution of 10%, 15%, 20%, 25%, 30%, and 35% sucrose were sequentially added to a sterile 15 mL centrifuge tube, and then 2 mL of protoplast solution was slowly added on top. After they were centrifuged at 50× *g* for 15 min, the protoplasts were harvested from the bands formed in each layer and observed under a microscope. (320×).

To determine the best sucrose concentration to be applied to the sucrose cushion method, a gradient experiment was performed with sucrose concentrations of 10% to 35% (Figure 3). The protoplast densities were 4.95 ± 1.06 × 10^5^ protoplasts/g FW, 4.80 ± 0.42 × 10^5^ protoplasts/g FW, 5.20 ± 0.85 × 10^5^ protoplasts/g FW and 0.35 ± 0.21 × 10^5^ protoplasts/g FW in response to sucrose concentrations of 10%, 15%, 20%, and 25%, respectively. Intact protoplasts were observed at 10–20% sucrose concentrations, and no viable protoplasts were found at 30% or 35% sucrose concentrations (Figure 3). According to these results, it was suggested that performing the sucrose cushion method with a sucrose concentration of 20% is the best method when considering protoplast concentration and viability. The established method can be summarized as follows: 2 mL of protoplasts was slowly added to 6 mL of 20% sucrose solution, after which the mixture was centrifuged at 50× *g* for 15 min.

### 2.4. Establishment of Conditions for Microcallus Induction

**Figure 4:**
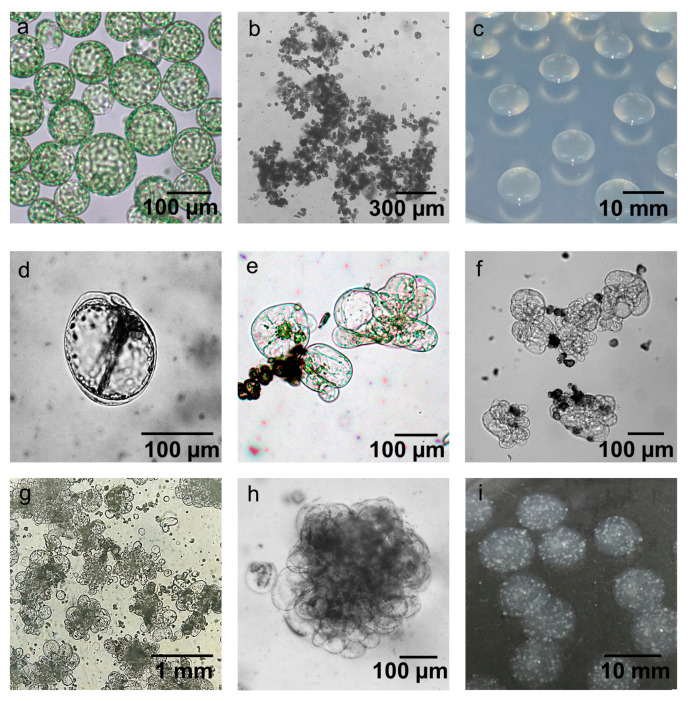
Induction of microcalli from potato protoplasts using an alginate lens. (**a**) Purified individual protoplasts. (**b**) Agglutination of protoplasts through liquid culture. (**c**–**i**) Fixation of protoplasts in alginate lenses, (**c**) and culture for 3 days (**d**), 5 days (**e**), 7 days (**f**), 2 weeks (**g**,**h**) and 3 weeks (**i**) for microcalli induction.

In protoplast-derived plant regeneration systems, the formation of microcalli is one of the most important early stages. Using high-quality protoplasts secured through established purification methods (Figure 4a), the effect of protoplast fixation through alginate lenses on micro callus formation was analyzed. Microcalli were rarely observed in treated cultured protoplasts without alginate fixation after one week of culture (data not shown). In addition, agglutination among protoplasts was frequently observed within one week (Figure 4b). When the protoplasts were fixed on the alginate lens (Figure 4c), the cell wall recovered after 3 days of culture, homogeneous division of the protoplasts started (Figure 4d), and the protoplasts developed extensively from the fifth day of culture (Figure 4e,f, seventh day of culture). Importantly, the cell walls of the protoplasts exposed to light or under microscopy observations were not reconstructed within the first 3 days of induction, resulting in no microcallus development. Approximately 2–3 weeks after protoplast culture, the microcalli formed in alginate lenses were visibly detected (Figure 4g–i).

Two different types of media were compared to determine the effects of protoplasts on the formation of microcalli. As shown in Table 4, the composition of the two media used differs mostly in terms of inorganic salts and vitamins. Compared with CI2 medium, CI1 medium, based on the study of Hunt and Helgeson [26], contains twice as many microelements and carbohydrates (excluding glucose, mannitol, and sucrose) and substantially fewer vitamins. In addition, there was no bovine serum albumin (BSA). Protoplasts showed better microcallus induction (18.2%) in CI2 medium than in CI1 medium (Table 5); the values were 6.26 ± 0.75 for CI1 and 7.40 ± 0.50 for CI2. Although the results for the CI2 medium were good, there was no statistical significance (*p*-value > 0.05, Student’s *t*-test) with respect to either medium (Table 5). Nevertheless, compared with those induced under CI1 medium, microcalli induced under CI2 medium proliferated and developed more vigorously under continuous culture for minicalli.

### 2.5. Appropriate Space Is a Key Factor for Minicallus Development

**Figure 5:**
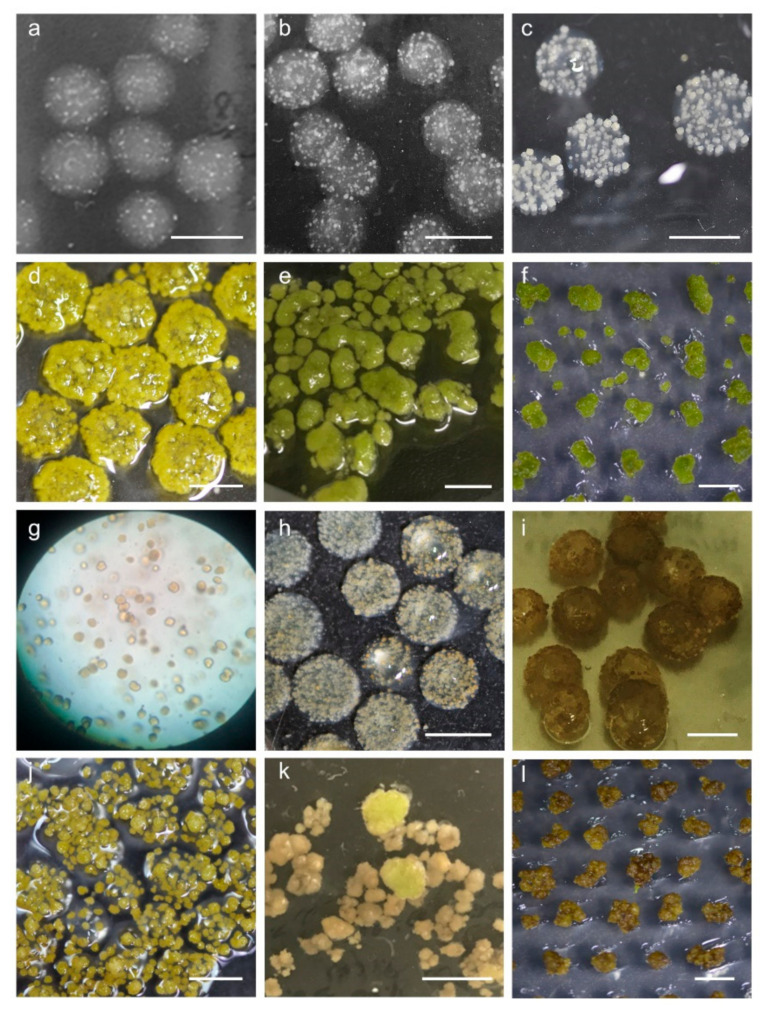
Images of microcallus induction to plant regeneration of potato protoplasts. (**a**,**b**) Initiation of microcalli from protoplasts in alginate lenses in CI medium for 2 weeks (**a**) and 3 weeks (**b**). (**c**,**d**) Development of minicalli in proliferation medium at 1 week (**c**) and 4 weeks (**d**). (**e**) Greening of alginate-released calli in G medium for 6 weeks. (**f**) Induction of regeneration of green calli in SI2 medium. (**g**–**l**) Conditions of calli for which the induction of plant regeneration from protoplasts is difficult. (**g**) Contaminated protoplasts. High-density protoplasts in an alginate lens. Development of minicalli from microcalli for 1 week (**h**) and 4 weeks (**i**) in P medium. (**j**) Low growth of minicalli in P medium for 4 weeks. (**k**) Low growth and greening of calli in SI1 medium for 6 weeks. (**l**) Browning of calli. Scale bar: 10 mm.

Microcalli in alginate lenses were incubated for 4 weeks in proliferation (P) medium to develop minicalli (Figure 5a–d). When protoplasts were contaminated with bacteria, all protoplasts in alginate did not develop into microcalli (Figure 5g). Therefore, when extracting protoplasts from leaves, it is necessary to check for the presence of endogenous bacteria in the leaves, and sterile in vitro plants should be used for extracting protoplasts. When a microcallus developed into a minicallus within an alginate lens, it also was found that obtaining appropriate space was an important factor. In other words, if 4.0 × 10^3^ protoplasts/alginate lenses were fixed to an alginate lens, a large amount of microcalli would form, but they would not grow properly and would display browning (Figure 5h,i). On the other hand, even though sufficient space (4.0 × 10^2^ protoplasts/alginate lens) for minicallus growth in the alginate lens was provided, low growth of minicalli was observed (Figure 5j). According to these results, fixing 2.0 × 10^3^ protoplasts in a single alginate lens allowed the protoplasts to continue to develop. The microcallus induced in CI1 medium presented a lower growth rate than the microcallus induced in CI2 medium when cultured in P medium for 4 weeks.

### 2.6. Callus Greening Affects on Shoot Regeneration

**Figure 6:**
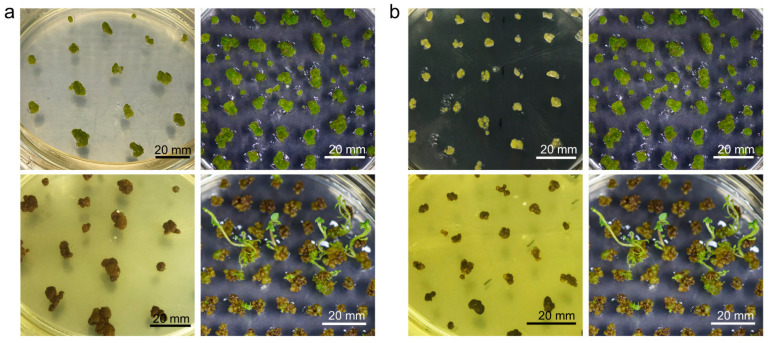
(**a**) Comparison of shoot regeneration from green calli in two different types of media. The upper panels show green calli over 5 mm in size in SI1 medium (left panel) and SI2 medium (right panel). The lower panels show the same calli as those in the upper panels after 1 month. (**b**) Comparison of shoot regeneration from green calli of different sizes in SI2 medium. The upper panels show green calli 2–5 mm in size (left panel) and over 5 mm in size (right panel). The lower panels show the same calli as those in the upper panels after 1 month.

Not all minicalli can be regenerated; the next step, greening, is more decisive. The greening of calli, which is the stage immediately before shoot regeneration, was induced in G medium (Table 4) for 4–6 weeks. After 4 weeks, the color of most of the viable calli with a sufficient size of 1 cm^2^ changed from light green to dark green (Figure 5e,f). To induce shoot growth from these green calli, the cells were transferred to regeneration (SI) medium. The SI medium essentially consisted of the PGRs naphthylacetic acid (NAA), gibberellin (GA_3_), and zeatin. SI1 medium was prepared according to the method previously established in our laboratory for the shoot induction of potatoes [27]. In terms of differences, compared with SI1 medium, SI2 medium contained half the amount of sucrose and lacked vitamins (Table 4). Despite SI1 medium having the same NAA, GA_3_, and zeatin contents as SI2 medium, no shoots were induced in the former, and the calli turned brown (Figure 6a). On the other hand, in SI2 medium, shoots were strongly induced from green calli after one month. We also confirmed that the method of transferring calli onto SI2 medium had a significant effect on regeneration efficiency. When the green calli were transferred to SI2 medium, active proliferation was observed in whole calli transferred without separation, whereas when calli were separated and divided into small sizes of 0.2–0.5 cm^2^, most calli browned and no longer developed (Figure 6b).

**Figure 7:**
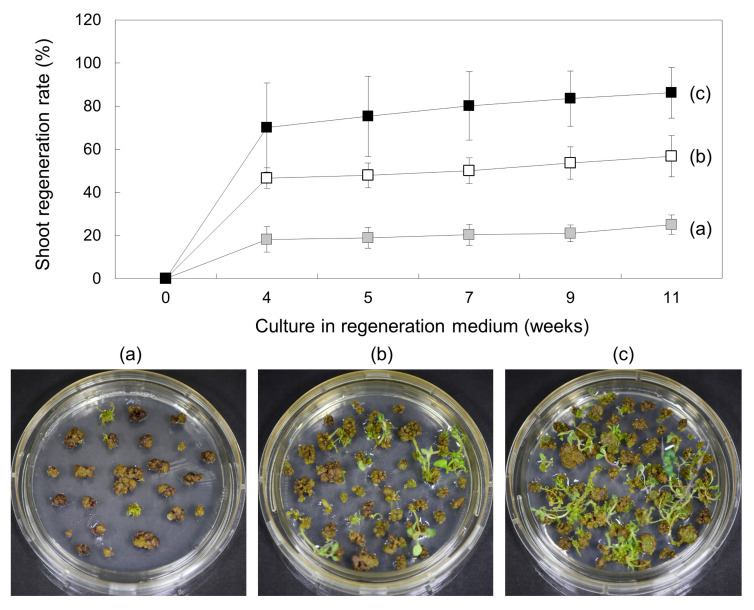
Effects of culture period on callus greening in G medium. Proliferated minicalli in P medium were cultured in G medium for 4 weeks (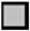, (**a**)), 5 weeks (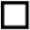, (**b**)), and 6 weeks (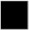, (**c**)) and then transferred to SI2 medium for shoot induction. Regeneration efficiency was measured using an average of 60 calli clumps per replicate. Three to five replicates were averaged. Error bars indicate ± standard deviation of the mean (SD).

The culture period for callus greening also affects shoot regeneration. Calli were cultured under the same conditions in G medium for 4 weeks, 5 weeks, and 6 weeks for greening. Initially, the regeneration rates of green calli for 4 weeks, 5 weeks and 6 weeks were 18.24 ± 5.88%, 46.63 ± 4.72%, and 70.18 ± 20.67%, respectively, but the rates increased as the culture progressed; in particular, the regeneration rate of calli that had undergone greening for 6 weeks reached 86.24 ± 11.76% at 11 weeks (Figure 7). This efficiency was approximately 3.3-fold higher than that of calli that had been greening for 4 weeks. Taken together, these results indicate that the degree of callus greening has a significant effect on regeneration efficiency.

## 3. Discussion

A reproducible and robust method of protoplast-derived plant regeneration is essential for the application of protoplasts in plant breeding, especially with respect to new plant breeding techniques such as clustered, regularly interspaced, short palindromic repeat (CRISPR) genome editing. In proportion to the importance of potato, many studies have been performed on protoplast isolation, culture, and plant regeneration methods. The protoplast extraction efficiency has recently been reported to be 1.9 × 10^6^ protoplasts/g FW leaf [28], which is similar to the results of previous research, in which values of 1.6 × 10^6^ protoplasts/g FW [29] and 2.4 × 10^6^ protoplasts/g FW were reported [30]. We performed our experiments according to the latest methods reported by Nicolia et al. [28]. As part of a preliminary experiment, an average yield of 1.23 ± 0.34 × 10^6^ protoplasts/g FW (M1 condition, 18 h) was obtained. Although these average results have been successfully reproduced, there is a need to develop a method that is more accessible, time-saving, and efficient. As such, we changed the leaf material preparation for wounding, extraction processes such as shaking and washing, the compositions of the media, and so on. Additionally, we skipped the pretreatment step, which is designed for addressing loose cell walls or plasmodesmata, because there was no significant difference between preincubation of the plant material or lack thereof (data not shown). On the other hand, various pretreatments, such as 24 h of incubation, dark pretreatment, and preincubation with Ca^2+^ have been shown to increase protoplast isolation [28,30,31]. In some plant species, pretreatment using antioxidants is needed to reduce phenolic compound accumulation and thus improve protoplast isolation [32]. In the case of in vitro-grown plants like the materials we used, it is thought that pretreatment may have little effect because there are few phenolic compounds present.

Plant materials using cotyledons (7–9 days old) and first true leaves (15–17 days old) for enzymolysis were compared by Huang et al. [33], and the results showed that, compared with cotyledons, true leaves underwent slightly less enzymolysis. Additionally, leaves of plants under drought or severe temperature stress are considered difficult materials to use for protoplast enzymolysis. Therefore, leaves of in vitro-grown plants are usually ideal materials for protoplast preparation. To verify the effects of the physiological state of the donor plant, in vitro-grown plants aged 2 weeks, 3 weeks, and 4 weeks, which were apically sub-cultured every 2 weeks, were used. In our study, the best efficiency of protoplast extraction was achieved under conditions (L3) in which axillary buds induced 1 week after node culture were sub-cultured in fresh MS medium for 2 weeks. The corresponding values differed by up to two times compared to the lowest values obtained for 4-week-old plants (Figure 2). Young leaves from 3–4-week-old in vitro shoots are mostly used for the protoplast extraction of potato [29,30], but plants older than 4–5 weeks are also sometimes used [28]. Otherwise, those older than 5 weeks exhibited very poor yields [34]. It is known that the cell walls of old plants become more rigid and difficult to dissolve, and mesophyll cells lose their ability to produce protoplasts [34,35].

In the protoplast isolation process, the enzymolysis time and concentrations of digestive enzyme mixtures directly affected the efficiency and viability. To increase protoplast release, a longer enzymolysis time is beneficial. Otherwise, continued enzymolysis could lead to protoplast breakdown and a consequent decrease in protoplast viability [36,37,38]. However, this depends on the tissue source. For example, even in the same plant, the enzymolysis time was 7 h when protoplasts were extracted from callus tissues [39], whereas 9 h was effective for protoplasts extracted from cotyledons [40]. The appropriate combination of enzyme solution and enzymolysis time influenced protoplast yield and viability.

In our study, the protoplast yield increased as the digestion time increased to 18 h, but with further increases in digestion time, protoplast yield decreased significantly (Table 2). We tried to shorten the time to identify a more efficient method, even though we obtained a result of 14.20 ± 2.96 × 10^5^ protoplasts/g FW. As a result, it was possible to reduce the time to 12 h by changing the extraction conditions, including the washing buffer volume, the types of enzymes used, and other factors.

The purification of protoplasts is an important process that can affect the extraction yield of high-quality protoplasts. Establishment of an appropriate protoplast purification method can suppress the reduction in protoplast extraction efficiency due to poor recoveries, and protoplasts purified in the presence of the enzyme in the crude protoplast releasing enzyme solution enable stable and viable protoplast maintenance. In addition, it can reduce the production of polyphenols from damaged protoplasts for protoplast division [41]. For these reasons, it is possible to secure viable protoplasts by selectively removing damaged protoplasts and other cellular debris using a sucrose gradient [42]. Previously reported protoplast purification in potatoes was performed using 15% sucrose [28]. In our results, the proportion of intact viable protoplasts was high under the purification conditions using 15% sucrose (~89%, Figure 3). However, even in the 15~20% sucrose layer, more than 75% of protoplasts were viable, and division from the purified protoplasts using 20% sucrose was also well tolerated. As a result, it was possible to obtain a greater number of intact viable protoplasts capable of protoplast division when purified using 20% sucrose.

Alginate has been reported to prevent the agglutination of protoplasts, support cell wall reconstruction, and promote mitotic division by reducing polyphenol production [41]. The stimulating effect of embedding protoplasts in sodium alginate has been demonstrated for many plant species, including apple [43], potato [44], and *Arabidopsis thaliana* [45]. The researchers of most of these studies used liquid culture for alginate-embedded protoplasts. However, we used solid media for culture and demonstrated that solidified alginate lenses are more efficient at inducing microcallus formation than liquid culture. Carrot protoplasts immobilized in alginate and cultured in liquid media successfully initiated mitotic division and formed cell colonies in 14 days [46]. In 1986 [29], potato protoplasts were shown to develop well in agarose media; additionally, the researchers tried to add fresh media to avoid protoplast aggregation, which resulted in the formation of white colonies and their separation. Nevertheless, 1.8% agarose was used as a mixing agent with protoplasts in other studies [47].

According to Andersson et al. [48], shoot regeneration is inhibited for protoplasts at excessively low and high densities. This means that an appropriate balance of protoplast concentration is needed. In this experiment, to culture as many individual protoplasts as possible, the culture was maintained at a density of 4 × 10^2^ protoplasts/alginate lens; however, this density was too low for the advancement of callus development and plant regeneration. In addition, minicallus induction from high-density microcalli in alginate lenses in proliferation medium prevented the formation of minicalli of sufficient size (Figure 5h,i). Appropriate plating densities of 1.0 × 10^5^ protoplasts/mL, which is one of the tested densities (0.5 × 10^5^ protoplasts/mL, 1.0 × 10^5^ protoplasts/mL, 1.5 × 10^5^ protoplasts/mL, and 2.0 × 10^5^ protoplasts/mL), proved efficient for the enhanced mitotic division of protoplasts [49]. Most of the growth-inhibited calli were brown when shoot growth was induced in the regeneration medium (Figure 5j–l).

The shoot regeneration response to PGR treatment varies according to cultivar. For example, cultivar Spunta can be started on regeneration medium containing zeatin and NAA, while cultivars Kennebec, Primura, and Desiree can be started on media containing benzylaminopurine (BAP) and GA_3_ [29]. Here, the PGR composition for shoot regeneration was 0.01 mg/L NAA, 2.0 mg/L zeatin, and 0.1 mg/L GA_3_. However, even though the PGR compositions of the two SI (SI1 and SI2) media used to induce regeneration from callus were the same, shoot growth was induced only in SI2 medium. Considering that SI1 medium is a medium specifically designed for in vitro shoot regeneration from leaf-derived calli [27], the results suggest that the regeneration requirements for calli derived from organogenesis from wounds of potato leaves and protoplast-derived calli differ. It is presumed that the hormone conditions required for plant regeneration differ between protoplast-derived calli and leaf tissue-derived calli, and that other substances, such as gelling agents in the media, also affect regeneration. Considering the phenomenon in which low concentrations of Gelrite had a positive effect on shoot regeneration, it should be considered that the type of agar in the medium may also be an important factor in protoplast-derived plant regeneration.

Protoplast-derived calli are known to induce shoot growth after callus greening [50]. It has been reported in several papers that light green calli first change to dark green calli, after which the shoots then differentiate; however, when light green calli turn brown, shoots no longer develop [47,51]. Callus greening was also found to be one of the most important factors for regeneration in this work. In the Solanaceae family, a combination of indoleacetic acid (IAA) and zeatin was used for plant regeneration from leaf protoplasts [52]; however, callus greening was induced in G medium containing IAA and zeatin in our study. The induction of callus greening increased with lengthening incubation period in G medium, and the regeneration efficiency of callus-induced greening for 4 to 6 weeks was compared in SI2 medium containing PGRs, which are known to play positive roles in the shoot development of potato plants. According to the results, regeneration from calli for which greening was induced for 6 weeks in G medium yielded a total efficiency of 86.24 ± 11.76% for 11 weeks in SI2 medium (Figure 7). The PGR effect on shoot formation is not always consistent and is cultivar- or experimenter-dependent. Zeatin or thidiazuron (TDZ) exerted inhibitory effects on shoot regeneration in studies by Guri et al. [51] and Perales and Schieder [43], but had positive effects in studies by Rahmani et al. [53], Grzebelus et al. [46], and Hassanein and Dorion [54]. In our experiment, the regeneration efficiency was different even under the same hormone conditions. Generally, sucrose, a representative carbon source required for plant growth, is known to increase growth and development as its concentration increases, but high concentrations of sucrose can decrease growth and development [55]. According to van Rensburg et al., the appropriate sucrose concentrations for regeneration were all different according to the genotypes of *Lachenalia* [56]. In particular, plant regeneration from protoplast-derived calli was affected by the concentration of sucrose, and regeneration efficiency was high (up to ~10 g/L sucrose concentration), but regeneration was reduced at higher concentrations in Lily [57]. In addition, regeneration from potato protoplasts was also performed at a concentration of 10 g/L sucrose [28]. These means that PGRs exert no significant effect on regeneration efficiency, and other additional factors, such as the concentration of sucrose, gelling agents, and vitamins, must be supported. In line with our results, several reports have demonstrated that shoot induction is better in medium solidified with Gelrite than with agar [43,54]. Protoplast-derived potato regeneration efficiency is known to be 20–30% for the cultivars Russet Burbank [58] and Delaware [47], while it is reported to be 43% for the cultivar Kuras [48], although transfection was involved. Recently, a regeneration efficiency of 63.88% was reported for cultivar Desiree [59]. Our results are significantly higher, with reported efficiencies of 86.24 ± 11.76% for cultivar Desiree, which means that there is still room for efficiency improvement by applying various advanced methods.

In this study, we investigated a protoplast-derived potato regeneration method that was more efficient and timesaving than a pre-established method; our method yielded more than 80% regeneration efficiency for in vitro-grown shoots. This system could be applied to rapidly developing genome editing techniques to help develop new potato cultivars.

## 4. Materials and Methods

### 4.1. Plant Growth Conditions

Sterilized potato (cultivar Desiree) shoots were cultivated on medium consisting of Murashige and Skoog (MS) salts and vitamins [60], 30 g/L sucrose, and 8 g/L plant agar, pH 5.7, based on an established in vitro tissue culture system [61,62]. The in vitro shoots were sub-cultured every two weeks, and the cultures were kept in a growth chamber at 25 °C under light provided by cool-white fluorescent lamps (photosynthetic photon flux = 60 umol/m^2^/s) with a 16 h photoperiod.

To analyze the effect of protoplast extraction efficiency with respect to the physiological status of plant materials, a total of four plant growth conditions were compared. The apical bud containing the 1st–2nd nodes was cut out and sub-cultured in new MS medium, and then protoplasts were extracted from in vitro-grown plants for 2 weeks (L1), 3 weeks (L2), and 4 weeks (L4). In addition, the 1st–3rd nodes (from which apical buds were removed) were cut out and transferred to MS medium to induce axillary buds. After about 1 week, the induced axillary bud was sub-cultured in a new MS medium for 2 weeks (L3) to extract protoplasts from the leaves of plants grown in vitro.

### 4.2. Preparation of Materials for Protoplasts

The pH of the media and stock solutions used (Table 6) was adjusted to 6.0. Both the enzymes for protoplast isolation and plant growth regulators (PGRs) were filter sterilized. All macro- and micro-elements, sucrose, and agar for tissue culture media were purchased from Duchefa (Haaelem, Netherlands), and other chemicals were purchased from Sigma-Aldrich (St. Louis, MO, USA). Cellulase ‘Onozuka R10′ and macerozyme were purchased from Yakult Pharmaceutical Co., Inc. (Tokyo, Japan).

### 4.3. Protoplast Extraction and Purification

The leaves at the 2nd–5th nodes from the top were removed from in vitro-grown plants that had been growing for 2 weeks. The leaves were 1.2 × 0.8 cm^2^ in size (average of 25 mg), and approximately 40–50 leaves were used at a time. For the extraction of protoplasts, the leaves were wounded using three different methods (Figure 1): peeling off (a), gently chopping (b), and forming leaf strips (c). The peeling off method was performed according to a modified version of the ‘tape–*Arabidopsis* sandwich’ method [63]. Briefly, both sides of the epidermis were adhered to clear 3M tape, and the lower epidermal layer was removed by carefully pulling away the 3M tape. These peeled leaves were then transferred to an enzyme solution. The second method, which is the most commonly used method, involved gently chopping the leaves into approximately 0.2 × 0.7 cm^2^ segments. Last, partial wounds involving 1–2 mm strips on both sides of the midrib were made with a sterile blade. The wounded leaves for digestion were soaked in 30 mL of extraction solution (E1) (Table 4) at room temperature (RT) in darkness with shaking at 50 rpm for 5 h. Subsequently, the protoplast suspension was filtered through a 75 μm sterile nylon mesh sieve, and a 30 mL wash (W) solution (Table 4) was passed through the sieve to recover the protoplasts from debris remaining in the sieve. Then, the solution containing the protoplast which passed through the sieve was centrifuged at 50× *g* for 5 min. The supernatant was carefully discarded, and the protoplast pellet was gently dissolved and washed twice with W solution, and then resuspended in 2 mL of W solution.

### 4.4. Protoplast Extraction from Enzymolysis Conditions

To analyze the efficiency of protoplast extraction from enzymolysis conditions, we tried to determine the effect of enzymolysis time. About 1 g of the leaf with induced partial wounds was soaked in 30 mL extraction solution with 50 rpm shaking in the dark for 5 h, 6 h, and 18 h, and then purified through sucrose cushioning to measure the density of protoplasts. In addition, in order to compare the effects on extraction according to the concentration of enzyme, extraction solutions of cellulase/macerozyme at concentrations of 1%/0.2% (Table 3, M1 condition) and 1%/0.5% (Table 3, M2 and M3 condition) were used for protoplast extraction without changing any other conditions. Furthermore, in order to confirm the extraction efficiency by the inhibition of enzyme activity during purification of the protoplast released by the enzyme solution, the protoplasts released from the extraction conditions (Table 3, M2 and M3 conditions) containing cellulase/macerozyme (1%/0.5%) were used in the extraction process. The released protoplasts were purified using the same volume of wash solution (Table 3, M2 condition) or a 3-fold volume of wash solution (Table 3, M3 condition) as the extraction solution.

### 4.5. Protoplast Purification by Sucrose Gradient

Experiments were conducted to establish the appropriate sucrose gradient assay conditions for the efficient purification of intact protoplasts from potato leaf extracts. Two milliliters of each solution of 10%, 15%, 20%, 25%, 30%, and 35% sucrose, respectively, were slowly layered to gradient layers on a sterile 15 mL centrifuge tube. A supernatant containing 2 mL of unpurified protoplasts was layered on top. The tube containing the solution divided into seven layers was centrifuged at 50× *g* for 15 min using a swing bucket centrifuge (minimum acceleration and deceleration). The solutions were harvested from the green-colored thin bands formed in each of several layers, and the intact protoplasts were observed under a microscope.

The density of protoplasts was determined by counting the number of protoplasts with a hemocytometer (Fuchs–Rosenthal hemocytometer chamber) under a microscope. For counting the protoplasts, 10 µL of the protoplast suspension was gently transferred to the chamber. The chamber was subsequently covered with a cover slip, after which the protoplasts within a 10 × 10 objective were counted. Protoplasts within several 1 mm^2^ squares were counted, and the average number per square millimeter was calculated. The protoplast concentration was calculated as follows: number of protoplasts in a 1 mm^2^ square × 1 × 10,000 = number of protoplasts/mL. On the basis of microscopy observations, we considered protoplasts that had perfect spherical shape and an intact membrane, and all the quantified protoplasts in this study were viable. The protoplast yield was measured as the total number of protoplasts divided by the fresh weight of the tissue used for protoplast isolation (protoplast/g FW).

### 4.6. Immobilization of Protoplasts in an Alginate Lens

The protoplasts and a sodium alginate solution consisting of 2.8% (*w*/*v*) alginic acid sodium salt and 0.4 M sorbitol were suspended at the same ratio (*v*/*v*) to a final concentration of 5 × 10^4^ protoplasts/mL, after which they were mixed by gently inverting the tubes. The mixed solutions were carefully poured onto agar consisting of 0.8% (*w*/*v*) phytoagar, 50 mM CaCl_2_·H_2_O, and 0.4 M sorbitol to form alginate droplets. After 2 h at RT, the droplets solidified into a translucent lens shape, which we refer to as an ‘alginate lens’. The alginate lenses were removed from the agar and then subjected to the subsequent steps.

### 4.7. Protoplast Culture and Plant Regeneration

Callus and shoot regeneration was performed by modifying a previously described method [26]. To induce microcalli (1–2 mm diameter) from protoplasts, the solidified alginate lenses were transferred to callus induction (CI1 and CI2) media (Table 4) and cultured in the dark at 24 °C. The resulting microcalli were then transferred to proliferation (P) medium (Table 4) and cultured for 4 weeks at 24 °C under light provided by cool-white fluorescent lamps (photosynthetic photon flux = 60 μmol/m^2^/s) with a 16 h photoperiod; the medium was replaced each week. To separate the calli from alginate lenses, 2 mL of releasing solution (consisting of 20 mM sodium citrate and 0.5 M sorbitol) was applied for 10 min, after which the calli were washed several times with P medium to remove alginate debris. The calli released from the lens were incubated in greening (G) medium (Table 4) to induce callus greening, with subculturing performed each week for 4–6 weeks. The calli that turned green were transferred to shoot induction (SI1 and SI2) media (Table 4) to induce shoot formation, and they were sub-cultured every two weeks.

### 4.8. Measurement of Regeneration Efficiency from Green Calli

To measure the regeneration efficiency of protoplast-derived calli, shoots were induced from calli-induced greening in G medium for 4 weeks, 5 weeks, and 6 weeks in SI medium. One shoot was excised per calli clump, in which greening of about 5 mm size was induced, and the calli-induced shoot was removed to measure the regeneration efficiency of the calli. Regeneration efficiency was measured using an average of 60 calli clumps per replicate, and at least three replicates were performed. The regeneration efficiency was observed at intervals of 5 weeks, 7 weeks, 9 weeks, and 11 weeks, starting from 4 weeks when the shoot was first induced in SI medium. These experiments were independently conducted at least three times. Regenerated shoots were separated and grown in MS basal medium without PGRs.

### 4.9. Statistical Analysis

Statistical analysis between different groups was evaluated with Student’s *t*-test. At least three biological replicates were performed for each analysis. Quantitative data are expressed as mean ± standard deviation (SD). Student’s *t*-test was conducted in Excel.

## Figures and Tables

**Table 1:** Comparison of extraction efficiencies of protoplasts from leaves (1 g fresh weight) in response to three different wounding methods.

Method	Wound Type	Extraction Efficiency (×10^5^ Protoplasts/g FW)	*p*-Value (Student’s *t*-Test)		
			vs. W1	vs. W2	vs. W3
W1	Peeling off	4.45 ± 1.23	-	0.1820	0.0194
W2	Gentle chopping	7.14 ± 2.38	0.1820	-	0.0473
W3	Leaf strips	13.23 ± 2.80	0.0194	0.0473	-

**Table 2:** Comparison of extraction efficiencies according to the enzymolysis time.

Enzymolysis Time	Enzyme Concentration		Wound Type	Extraction Efficiency (×10^5^ protoplasts/g FW)	*p*-Value (Student’s *t*-Test)		
	Cellulase R-10 (%, *w*/*v*)	Macerozyme (%, *w*/*v*)			vs. 5 h	vs. 18 h	vs. 24 h
5 h	1	0.2	Leaf strips	5.24 ± 1.90	-	0.0165	0.0492
18 h	1	0.2	Leaf strips	14.20 ± 2.96	0.0165	-	0.0942
24 h	1	0.2	Leaf strips	9.38 ± 0.88	0.0492	0.0942	-

**Table 3:** Comparison of extraction efficiencies according to the combinations of various factors for protoplast extraction.

Conditions	Medium	Cellulase/ Macerozyme (%, *w*/*v*)	Volume of Wash Soln.	Shaking (rpm)	Plasmolysis Time (h)	Enzymolysis Time (h)	Extraction Efficiency (×10^5^ Protoplasts/g FW)
M1	20 mM 2-(N-Morpholino)ethanesulfonic Acid (MES) 0.5 M mannitol 20 mM KCl 10 mM CaCl_2_ 0.1% Bovine Serum Albumin (BSA) pH 5.7	1/0.2	1×	0	1	5	5.24 ± 1.90
						6	8.56 ± 1.84
						18	12.27 ± 3.40
M2	20 mM MES 0.5 M mannitol 20 mM KCl 10 mM CaCl_2_ 0.1% BSA pH 5.7	1/0.5	1×	40	0	5	21.09 ± 8.49
						6	39.63 ± 4.09
						18	28.14 ± 3.34
M3	20 mM MES 0.5 M mannitol 20 mM KCl 10 mM CaCl_2_ 0.1% BSA pH 5.7	1/0.5	3×	40	0	5	36.40 ± 4.66
						6	63.59 ± 5.14
						18	N/A

**Table 4:** Media used for protoplast culture.

Abbreviation	Basal Medium	Components	Purpose
E1		20 mM MES, 1% cellulase ‘Onozuka R10′, 0.5% macerozyme, 0.5 M mannitol, 20 mM KCl, 10 mM CaCl_2_, 0.1% BSA, pH 5.8 (autoclaved)	Protoplast extraction
W		Macro C and micro C stocks, iron elements C, 2.0 mg/L α-Naphthaleneacetic Acid (NAA), 0.5 mg/L 6-Benzylaminopurine (BAP), 14.03 g/L NaCl, pH 5.6	Protoplast washing
CI1		Macro B and micro B stocks, iron elements B, carbohydrates B, vitamins B, 1.0 mg/L NAA, 0.4 mg/L BAP, and other organics, pH 5.6	Microcallus induction
CI2		Macro A and micro A stocks, iron elements A, carbohydrate A, vitamins A, 1 g/L BSA, 1.0 mg/L NAA, 0.4 mg/L BAP, and other organics, pH 5.6	Microcallus induction
P	MS	107 mg/L NH_4_Cl, 2.5 g/L sucrose, 54.7 g/L mannitol, 40 mg/L adenine sulfate, 0.1 g/L casein hydrolysate, vitamins C, 0.1 mg/L NAA, 0.5 mg/L BAP	Proliferation
G	MS	267.5 mg/L NH_4_Cl, 2.5 g/L sucrose, 36.4 g/L mannitol, 80 mg/L adenine sulfate, 0.1 g/L casein hydrolysate, vitamins C, 0.1 mg/L IAA, 2.5 mg/L zeatin	Callus greening
SI1	MS	30 g/L sucrose, 0.01 mg/L NAA, 2.0 mg/L zeatin, 0.1 mg/L Gibberellic acid (GA_3_), 4.0 g/L plant agar, pH 5.6	Shoot regeneration
SI2	MS	10 g/L sucrose, no vitamins, no myo-inositol, 0.01 mg/L NAA, 2.0 mg/L zeatin, 0.1 mg/L GA_3_, 2.5 g/L gelrite, pH 5.6	Shoot regeneration

**Table 5:** Comparison of microcallus induction efficiencies between two different types of culture media (CI1 and CI2).

Microcallus Medium	Protoplasts/ Alginate Lens (A)	Callus Counts/ Alginate Lens (B)	Callus Induction Efficiency (%) (B/A × 100)
CI1	2 × 10^3^	125.13 ± 15.01	6.26 ± 0.75
CI2	2 × 10^3^	147.95 ± 10.06	7.40 ± 0.50

**Table 6:** Stock solutions for medium preparation.

**Macro-Elements**	**A (mg/L)**	**B (mg/L)**	**C (mg/L)**
KNO_3_	740	1900	740
MgSO_4_·7H_2_O	492	350	492
KH_2_PO_4_	34	680	34
CaCl_2_·2H_2_O	368	600	882
**Iron Elements**	**A**	**B**	**C**
Na_2_EDTA	14	37.3	14
FeSO_4_·7H_2_O	19	27.8	19
**Micro-Elements**	**A**	**B**	**C**
H_3_BO_3_	1.5	3	1.5
MnSO_4_·H_2_O	5	10	5
ZnSO_4_·7H_2_O	1	2	1
Na2MoO_4_·2H_2_O	0.12	0.25	0.12
CuSO_4_·5H_2_O	0.012	0.025	0.012
CoCl_2_·6H_2_O	0.012	0.025	0.012
KI	0.38	0.75	0.38
**Carbohydrates (g/L)**	**A**	**B**	
Glucose	33.7	10	
Mannitol	30.92	40	
Sucrose	0.125	10	
Sorbitol	0.125	0.25	
D(−)Fructose	0.125	0.25	
D(−)Ribose	0.125	0.25	
D(+)Xylose	0.125	0.25	
D(+)Mannose	0.125	0.25	
L(+)Rhamnose	0.125	0.25	
D(+)Cellobiose	0.125	0.25	
Myo-inositol	0.05	0.05	
**Vitamins**	**A**	**B**	**C**
Pantothenic acid	2.5	0.5	-
Choline Chloride	2.5	0.5	-
Ascorbic acid	5	1	-
p-Aminobenzoic acid	0.05	0.01	-
Nicotinic acid	2.5	5	5
Pyridoxine-HCl	2.5	0.5	0.5
Thiamine-HCl	25	2	0.5
Folic acid	1	-	0.5
Riboflavin	-	0.1	-
Biotin	0.025	0.005	0.05
Cyanocobalamin	0.05	-	-
Cholecalciferol (Vit. D)	0.025	-	-

## Data Availability

All the data are included in the present study.
